# Professional Dignity in France

**Published:** 1864-04

**Authors:** 


PROFESSIONAL DIGNITY IN FRANCE.—Our readers have not forgotten how certain parties have of late been summarily dealt with by the Medical Council and the College of Surgeons for unworthy conduct. A measure of the same kind has lately been adopted by the Academy of Medicine of Paris, respecting a medical man named Priou, practising at Nantes. This individual was a correspondent of the Academy, and had lately, at the Medical Congress of Rouen, bills posted all over the town with advertisements of the most reprehensible kind. The members of the Congress protested very energetically, and the Academy has at last taken up the matter. It has been decided that Priou, for his forgetfulness of professional dignity, should be erased from the list of correspondents.

